# Measuring access to contraceptive methods by ethnic groups in Colombia using small areas estimation

**DOI:** 10.1590/0102-311XEN038024

**Published:** 2025-06-27

**Authors:** Lina María Sánchez-Céspedes, Juan Sebastián Oviedo-Mozo, Juliana Guerrero, Carlos Arturo Ramirez-Hernandez

**Affiliations:** 1 Departamento Administrativo Nacional de Estadística, Bogotá, Colombia.; 2 Universidad Nacional de Colombia, Bogotá, Colombia.; 3 United Nations Population Fund, New York, U.S.A.; 4 Inter American Development Bank, Washington, U.S.A.; 5 Université de Montréal, Montréal, Canada.

**Keywords:** Contraception, Reproductive Rights, Ethnic Inequatity, Family Planning Services, Decision Making, Anticoncepción, Derechos Sexuales y Reproductivos, Inequidad Étnica, Servicios de Planificación Familiar, Toma de Decisiones, Anticoncepção, Direitos Sexuais e Reprodutivos, Desigualdades Étnicas, Serviços de Planejamento Familiar, Tomada de Decisões

## Abstract

Afro-descendant and Indigenous women face limited access to contraceptives due to contextual barriers, partner disapproval, and educational gaps. One major challenge in designing policies to facilitate access to safe, effective, acceptable, and affordable contraceptive methods for these groups is the lack of data for informed decision-making by national and local governments. To address this information gap, we propose using small area estimation (SAE) focusing on population and ethnic groups rather than geographic areas. We estimated four state-level indicators for women by ethnic group: unmet need for family planning, family planning need satisfied, contraceptive prevalence rate, and modern contraceptive prevalence rate. SAE yielded consistent estimates with mean square errors mostly below 1% of the estimated values at both state and ethnic group levels. Contraceptive prevalence rates among Indigenous, afro-descendant, and non-ethnic women (who do not self-identify with an ethnic group) were 70.6%, 76.6%, and 81.7%, respectively. Lower contraceptive use is not determined by ethnicity. Being in a relationship increases the likelihood of contraceptive use; however, when afro-descendant and Indigenous women have partners from the same ethnic background this probability decreases. Consequently, addressing disparities in contraceptive use among ethnic groups requires government initiatives involving both women’s partners and communities.

## Resumen

Las mujeres afrodescendientes e indígenas tienen un acceso limitado a los anticonceptivos debido a las barreras contextuales, a la desaprobación de la pareja y a las brechas educativas. Uno de los desafíos en el diseño de políticas para facilitar el acceso a métodos anticonceptivos seguros, efectivos, aceptables y asequibles entre estos grupos es la falta de información para la toma de decisiones informadas por parte de los niveles locales y federales de gobierno. Para llenar este vacío de información, proponemos utilizar la estimación en áreas pequeñas (EAP) centrada en la población y en los grupos étnicos en lugar de las áreas geográficas. Estimamos cuatro indicadores para las mujeres por estado y por grupo étnico: necesidad insatisfecha de planificación familiar, necesidad satisfecha de planificación familiar, tasa de prevalencia de anticonceptivos y tasa de prevalencia de anticonceptivos modernos. Obtuvimos estimaciones consistentes utilizando la EAP, con errores cuadráticos medios en su mayoría inferiores al 1% de los valores estimados tanto a nivel del estado como de grupo étnico. Las tasas de prevalencia de anticoncepción entre las mujeres indígenas, afrodescendientes y no étnicas (que no se identifican con un grupo étnico) fueron, respectivamente, del 70,6%, 76,6% y 81,7%. Encontramos que el menor uso de anticonceptivos no está determinado por el origen étnico. Se observó que estar en una relación aumenta la probabilidad de uso de anticonceptivos. Sin embargo, cuando las mujeres afrodescendientes e indígenas tienen parejas del mismo grupo étnico, lo cual es más habitual, la probabilidad de usar métodos anticonceptivos es menor. Por lo tanto, abordar las disparidades con el uso de anticonceptivos entre grupos étnicos requiere iniciativas gubernamentales que involucren tanto a las parejas de las mujeres como a las comunidades en las que se encuentran.

## Resumo

Mulheres afrodescendentes e indígenas possuem acesso limitado a contraceptivos devido a barreiras contextuais, desaprovação dos parceiros e lacunas educacionais. Um dos desafios na elaboração de políticas para facilitar o acesso a métodos contraceptivos seguros, eficazes, aceitáveis e acessíveis entre esses grupos é a falta de informações para a tomada de decisões informadas pelas esferas locais e federais de governo. Para preencher essa lacuna de informações, propomos a utilização da estimativa de pequenas áreas (EPA), focando em grupos populacionais e étnicos, em vez de áreas geográficas. Estimamos quatro indicadores para mulheres em nível estadual por grupo étnico: necessidade não atendida de planejamento familiar, necessidade de planejamento familiar atendida, taxa de prevalência de contracepção e taxa de prevalência de contraceptivos modernos. Obtivemos estimativas consistentes usando a EPA, com erros quadráticos médios majoritariamente inferiores a 1% dos valores estimados, tanto no nível estadual quanto no de grupos étnicos. As taxas de prevalência de contracepção entre mulheres indígenas, afrodescendentes e não-étnicas (que não se identificam com um grupo étnico) foram, respectivamente, 70,6%, 76,6% e 81,7%. Descobrimos que o menor uso de contraceptivos não é determinado pela etnia. Observou-se que estar em um relacionamento aumenta a probabilidade de uso de contraceptivos. No entanto, quando mulheres afrodescendentes e indígenas têm parceiros do mesmo grupo étnico, o que é mais comum, a probabilidade de uso de métodos contraceptivos é menor. Assim, abordar disparidades no uso de contraceptivos entre grupos étnicos requer iniciativas governamentais envolvendo tanto os parceiros das mulheres como as comunidades que estão inseridas.

## Introduction

Research highlights limited access and use of contraceptive methods among Indigenous and afro-descendant women due to contextual barriers, partner disapproval, and educational gaps ^1^ ^,^ ^2^ ^,^ ^3^ ^,^ ^4^, but have not been measured until now. In this regard, this article makes three key contributions to the existing literature. Firstly, it assesses the accessibility and usage of contraceptive methods by women from different ethnic backgrounds in Colombia, facilitating the analysis of disparities between ethnic groups and informing public policy development. Secondly, it identifies several factors that underlie ethnic disparities related to family planning. Thirdly, it demonstrates the broader applicability of small area estimation (SAE) beyond territorial boundaries, showcasing its utility in estimating various indicators for small populations or domains. 

Regarding this last contribution, Ghosh & Rao ^5^ ^,^ ^6^ clarify that while “small area” usually denotes a specific geographic location like a county, municipality, or census division, it can also refer to a “small domain”. This may encompass demographic subgroups, including specific age, gender, or ethnic categories within a broader geographic context ^5^ ^,^ ^6^ ^,^ ^7^. For example, the Institute of Medicine ^8^ in the United States has reported health indicators for various demographic subgroups, including racial and ethnic groups, age categories, and socioeconomic strata. The country’s National Agricultural Statistics Service (NASS) has used SAE to estimate average hourly wage rates for field workers, livestock workers, field and livestock workers combined, and all hired workers (including supervisors, managers and others) by state ^9^. Economic characteristics like different categories of manufacturers or manufacturing sectors can also be defined as domains ^10^.

An ethnic-specific statistical approach reveals diverse ethnic groups’ needs and living conditions, shedding light on these issues and serving as an information source for acknowledging population and cultural diversity, facilitating the design of initiatives that safeguard ethnic groups’ rights. In Colombia, one such initiative is the Policy on Sexual and Reproductive Rights, the validity of which ended in 2021 ^11^. Afro-descendant and Indigenous communities are expected to participate in planning and evaluating the new health model and the future National Policy on Sexual and Reproductive Rights ^12^. This new policy will be an opportunity to find common ground between the healthcare knowledge of afro-descendant and Indigenous groups and non-ethnic ones (non-ethnic refers to individuals who do not self-identify as belonging to a specific ethnic group). Thus, this new policy should consider diverse ethnic groups’ collective identity, cultural customs, language, feelings, and behaviour. We hope that the indicators estimated at ethnic levels here can support this new policy.

A limiting factor in designing policies and programs that enable access to safe, effective, acceptable, and affordable contraceptive methods for afro-descendant and Indigenous women worldwide is the lack of data for adequate decision-making among national and local governments ^13^. We propose using SAE on population groups rather than geographic areas to bridge this information gap. SAE analyses have usually been associated with the analysis of specific domains or areas when, in fact, the concept can be extended to small populations which are not necessarily concentrated in a particular territory but may be dispersed throughout a particular territory rather than concentrated. We have computed the following ethnic level indicators for married or in-union women (aged 13 to 49 years) ethnic level for all Colombian states: (1) contraceptive prevalence rate, (2) modern contraceptive prevalence rate, (3) proportion of women with unmet needs for family planning, and (4) proportion of women whose family planning needs are met with modern methods. 

Importantly, Colombia’s National Sexual and Reproductive Health Policy 2014-2021, which is based on the International Conference on Population and Development of the Cairo Action Document from 1994, states:

“*A good state of Sexual and Reproductive Health implies the ability to enjoy a satisfactory and risk-free sexual life, the possibility of exercising the right to procreate or not, the freedom to decide the number and spacing of children*” ^11^ (p. 51) *“…regardless of the person’s sex, age, ethnicity, class, sexual orientation or marital status, and taking into account their specific needs according to their life cycle*” ^11^ (p. 61).

However, Indigenous people might have different perceptions about sexual and reproductive health. For instance, the Profamila Association asked members of the Yukpa people about their opinion regarding the statement that maintaining pleasurable sexual relations is important for the sexual life and general well-being of women and men ^14^. Results showed that 53.4% of women and 52.2% of men neither agreed nor disagreed with this statement, suggesting that they did not see a correlation between pleasurable sexuality and well-being.

Given the particular cultures and traditions of afro-descendant and Indigenous communities in Colombia, we provide a brief context of their reproductive customs and general opinions on contraceptive use. This overview is based on the few studies that have been conducted on the topic.

Indigenous peoples accounted for about 2 million in 2018, representing 4.4% of the Colombian population. Fertility rates among Indigenous peoples tend to be higher than those of other demographic groups due to the unique characteristics, historical background, and social organisation of these communities. Based on 2018 census data, the estimated total fertility rate (TFR) for Indigenous women was 3.56 children per woman in rural areas and 2 children per woman in urban areas ^15^. These figures are higher than the national average TFR of 1.95 ^16^. Indigenous women typically start their sexual and reproductive lives with the onset of menarche, signalling their readiness to start a family and become mothers ^17^. When it comes to fertility regulation methods, some Indigenous communities reject contraception due to the associated negative stigma, as their cultural ideal centres around the continuous growth of their community ^2^.

In 2018, afro-descendant communities were 9.7% of the total population, totalling 4,671,160 inhabitants. Based on 2018 census data, the estimated TFR for afro-descendant women was 2.28 children per woman ^18^. Notably, 15.7% of afro-descendant women aged 15 to 19 years are mothers compared with 11.4% of non-ethnic women in the same age group. This trend persists among women ages 20 to 24: 49.3% of the former group are mothers against 40.6% of the latter group ^18^.

Since the afro-descendant population has intermingled more with non-ethnic groups than with the Indigenous communities, distinct and noticeable fertility patterns can be observed among it across the national territory, unlike among Indigenous peoples. For afro-descendant populations concentrated in major cities like Bogotá, Medellín, and Cali, their fertility rates fall below the replacement level of 2 children per woman ^18^. However, despite this lower fertility rates in urban areas, early fertility is an important concern. Afro-descendant women residing on the Pacific Coast, where most afro-descendant communities reside, tend to abstain from using any contraceptive methods due to cultural traditions passed down through generations. Moreover, their partners often do not permit contraceptive use ^1^. This cultural aspect contributes to higher fertility rates in these communities like the 2018 TFR of 2.84 in Chocó ^16^.

Aside from this introduction, this article is divided into five sections. The second section describes the data. The third section explains SAE implementation to estimate contraception use indicators. The fourth section analyses the results of the probabilistic models and compares them across ethnic groups and departments. The fifth section discusses the results, and the sixth section brings concluding remarks.

## Data

Data from the 2015 *Colombian Demography and Health Survey* (DHS) ^19^ and the most recent population and household census (2018) ^20^ were used in this analysis. The DHS collected data on topics related to sexual and reproductive health from a sample of a population who was agreed from 13 to 69 years in rural and urban areas across the 33 states in the country. The final sample size equalled 92,799 people, of which 52,479 were women and 40,300 men, representing 44,614 households ^21^. The 2018 census collected sociodemographic characteristics on the national population, which amounted to 44,164,417 people (51.2% women; 48.8% men). This study only considered married or in-union women who were aged from 13 to 49 years from both data sources due to the impossibility of identifying sexually active women in the 2018 census.

## Method

On one hand, the only survey in Colombia that provides detailed information about access to contraceptive methods or its use is the DHS. This survey has a representative sample at the state (or department in Colombia) level. Therefore, it is impossible to calculate family planning indicators directly for ethnic groups at this level. On the other hand, the 2018 census ignores questions related to the use of contraceptive methods. However, it is the only source with representativeness at the department and ethnic levels. For this reason, SAE is used to estimate these indicators for this population disaggregation by utilising the 2018 census and the 2015 DHS. To achieve this, an estimation is first done with the 2015 DHS (for example, the probability of using modern methods), and then the prediction is made using the census data. The predictor variables must be the same in both sources or as close as possible for this process to work. 

The proposed methodology aims to model the following indicators for small domains and ethnic groups:

(a) Use modern and traditional: contraceptive prevalence rate. 

(b) Use modern: modern contraceptive prevalence rate.

(c) Unmet need: proportion of women with unmet need for family planning.

(d) Need satisfied: proportion of women whose family planning needs are met with modern methods.

These are essential indicators for monitoring the sexual and reproductive rights that are part of the global agenda of the Sustainable Development Goals (SDGs) and the regional agenda for Latin America and the Caribbean of the Montevideo Consensus. To estimate these indicators by ethnicity for each Colombian state, we followed the following steps ^22^.

### Step 1: homologation of variables from the 2015 DHS and 2018 census

Since the predictor variables in the 2015 DHS and 2018 census must be the same or as close as possible to implement SAE, a process of homologating these variables between the two sources was carried out. Some categories of the variables differed between the DHS and the census. In such cases, a new variable was built in both databases, ensuring its categories were as similar as possible.

For example, the built wall material variable was named ‘No walls, dirt, bamboo with mud, cane, palm, trunks, tin, cardboard’, and it had two categories: 1 corresponds to the wall categories in the 2015 DHS that indicate inadequate materials (no walls, natural walls, cane/palm/trunks, dirt, cardboard, reused wood, uncovered adobe, bamboo with mud, or plywood). These categories are equivalent to those in the 2018 census representing similar conditions (guadua, rough wood, board, plank, cane, mat, and other vegetation, with no walls or waste materials). Similarly, 0 identifies walls built with good materials in both the 2015 DHS (finished walls: cement, stone with lime/cement, stone with mud, bricks, cement blocks, covered adobe) and the 2018 census (block, brick, stone, polished wood, poured concrete, rammed earth, bahareque, covered adobe, and prefabricated material).

### Step 2: model estimation with survey data

A probabilistic model was fit at the individual level with the 2015 DHS, one for each of the following indicators and binary response variables:

(a) Contraceptive prevalence rate:

$$y_{D6,ed,i}=\{1\;if\;she\;uses\;contraceptive\;methods,\;0\;if\;she\;does\;not\;use\}$$

(b) Modern contraceptive prevalence rate:

$$y_{D6M,ed,i}=\{1\;if\;she\;uses\;modern\;contraceptive\;methods,\;0\;if\;she\;does\;not\;use\}$$

(c) Proportion of women with unmet need for family planning:

$$y_{UNMET,ed,i}=\{1\;if\;she\;has\;unmet\;needs\;for\;contraception,0\;if\;she\;does\;not\;have\}$$

The probability of $y_{A,ed,i}=1$ , in which *A* may be “use modern and traditional”, “use modern”, or “unmet need” is $p_{A,ed,i}$ and is given by the following equation:

$$logit\;\left(p_{A,ed,i}\right)=ln\left(\frac{p_{A,ed,i}}{1-pA,ed,i}\right)=x_{ed,\;\;i}^{T}\beta_{A}+v_{A,ed}{+\varepsilon }_{A,ed,i}$$

In which *i* denotes each individual and *ed* the ethnic group *e* in the state *d*. These include three ethnic groups and 33 states. The binary response variable $y_{A,ed,i}$ is assumed to be independent Bernoulli ( $p_{A,ed,i}$ ). $p_{A,ed,i}$ , obeying the logistic linking model with random effects $v_{A,ed}∼N\left(0,\sigma_{A,ed}^{2}\right)$ with individual errors $\varepsilon_{A,ed,i}∼N\left(0,\sigma_{A,ed}^{2}\right)$ and $x_{ed,i}$ individual-specific covariates. 

$p_{A,ed,i}$ is modelled using mixed-effects models because women in an ethnic group usually interact in a context in which other people also have similar beliefs regarding contraception. Hence, we have a hierarchical system with two levels: women and the group of people with whom they interact. As explained in the *Introduction*, Indigenous communities often reject contraception because of the negative stigma attached to it as their cultural values emphasise the ongoing growth of their population. Afro-descendant communities also generally refrain from using contraceptive methods due to deeply rooted cultural traditions handed down across generations, with partners frequently prohibiting its use.

To better understand the structure of the data, we calculated the intraclass correlation coefficient (ICC), which quantifies the proportion of overall variability that is due to differences between groups. The ICC can assess how much of the unexplained variance is due to group-level factors. This highlights the importance of including random effects in the model to capture between-group variability. To estimate the confidence interval of ICC, we performed a bootstrap with 500 replicates. We found the following ICC for use modern and traditional, use modern and unmet need, respectively (mean, 95% confidence interval - 95%CI): 076 0.0476; 0.115, 0.07 0.0431; 0.110 and 0.058 0.0262; 0.101.

For feasibility reasons, we define these two levels as women and ethnic-group-state individuals since a smaller disaggregation, such as municipality, showed convergence problems probably because of the absence of observations for some sub-groups. Moreover, we found that a random effects model could grasp some of the variability the independent variables were unable to fully explain. The mixed-effects models were estimated using *glmer* from R (http://www.r-project.org), which uses maximum likelihood.

### Step 3: estimating the predicted indicator at the individual level using the 2018 census

The predicted probabilities of the three indicators ( ${\hat{p}}_{\;Use\;modern,ed,i}$ , ${\hat{p\;}}_{Use\;modern\;and\;traditional,ed,i}$ and ${\hat{p}}_{Unmet\;need,ed,i}$ ) were estimated at the individual level for married or in-union women ages 13 to 49 with the 2018 census utilising the estimated models of step 2. 

The proportion of women who have their need for family planning satisfied with modern methods was estimated by the following equation ^20^:

$${\hat{p\;}}_{Need\;satisfied,\;ed,i}=\frac{{\hat{p\;}}_{Use\;modern,ed,i}}{{\hat{p}}_{\;Use\;modern\;and\;traditional,ed,i}+{\hat{p}}_{\;Unmet\;need,ed,i}}$$

### Step 4: estimating the predicted indicator at the small area level

To ensure that the ethnic figures estimated with the census at the national and department levels are consistent with the figures reported by the 2015 DHS at the national, state, rural, and urban levels, we applied Benchmarking to obtain the weights in the census that make this possible. To do this, we have the following information:

${\hat{p}}_{A,ed,i}$ : estimate for indicator *A* (use modern and traditional, use modern, unmet need, need satisfied) in the census for individual *i*. Since the random effect was already considered to estimate; ${\hat{p}}_{A,ed,i}$ to simplify notation ${\hat{p}}_{A,ed,i}={\hat{p}}_{A,i}$ .

$D_{A,j}$ : a direct estimate of indicator *A* calculated with the 2015 DHS and its weights for the disaggregation level *j*, in which *j* can be national, state, rural, and urban levels. These levels have been reported in the 2015 DHS document ^21^. The adjusted ratio *R*_*j*_ for each level *j* is given by ^22^ ^,^ ^23^:

$$R_{j}=\frac{\hat{D_{A,j}}}{\frac{1}{N_{j}}\sum_{1}^{N_{j}}{\hat{p}}_{A,i}}=\frac{\hat{D_{A,j}}}{\hat{Y_{A,j}}}$$

*N*_*j*_ is the number of individuals in the j level and $Y_{A,j}$ is the estimate of indicator *A* with the census for disaggregation level *j*. This ratio adjusts the census estimate to the one reported by the DHS. However, since *R*_*j*_ is different for each *j*, the goal of the Benchmarking algorithm is to determine a set of weights for the individuals in the census that achieves the *R*_*j*_ values for all *j*. Therefore, these weights ensure that the indicators estimated at the national, state, rural, and urban levels using the census align with those from the DHS.

### Step 5: mean square error estimation

The mean square errors are calculated using Bootstrap following the steps described by Rao & Molina ^24^.

## Results

This section has two parts. The first section interprets the results of the probabilistic models in step 2. The second section analyses the estimated indicators at the ethnic and state levels in step 4 (Supplementary Material ,https://cadernos.ensp.fiocruz.br/static//arquivo/supl-e00038024_9613.pdf) reports the estimated values of the four indicators at state levels by ethnic group and their mean square errors in step 5.

### The models on the 2015 DHS

Table 1 shows the results of the probabilistic models. Regarding age, we included five-year-age dummy variables, and the comparison category included women who were aged from 45 to 49 years. Results show that, maintaining all variables except age at a fixed value, the odds of using any contraceptive method, modern or otherwise, tend to increase over age up to 34 years. After this age, there occurred a decrease in the odds ratios (OR). This is consistent with the literature regarding naturally diminished fertility due to the transition to menopause and personal life decisions after age 34 years ^25^ ^,^ ^26^ ^,^ ^27^. We found an inverse relationship between age and the probability of unmet needs. The youngest group, women aged from 13 to 14 years, showed the highest OR, followed by those aged from 15 to 19 years and those aged from 20 to 24 years. The respective OR for these age groups totalled 7.06, 2.5, and 1.66.

**Table 1:** Probabilistic models on family planning methods.

	Using any contraceptive method = 1/otherwise = 0		Using a modern contraceptive method = 1/otherwise = 0		Having unmet needs = 1/otherwise = 0		
	Coefficient (SE)	OR	Coefficient (SE)	OR	Coefficient (SE)	OR
Intercept	-3.538 (0.373) *	0.029	-3.711 (0.357) *	0.024	-0.384 (0.409)	0.681
Aged from (years)						
13 to 14	0.309 (0.515)	1.362	0.359 (0.511)	1.432	1.955 (0.499) *	7.063
15 to 19	0.815 (0.108) *	2.259	0.737 (0.102) *	2.089	0.918 (0.150) *	2.504
20 to 24	0.927 (0.081) *	2.526	0.932 (0.075) *	2.540	0.508 (0.123) *	1.661
25 to 29	0.837 (0.073) *	2.310	0.834 (0.067) *	2.301	0.005 (0.120)	1.005
30 to 34	1.023 (0.075) *	2.781	0.888 (0.067) *	2.430	-0.335 (0.125) *	0.715
34 to 39	0.790 (0.074) *	2.204	0.685 (0.066) *	1.984	-0.255 (0.125) **	0.775
40 to 44	0.731 (0.077) *	2.077	0.605 (0.069) *	1.831	-0.222 (0.126) ***	0.801
Mother	0.688 (0.022) *	1.990	0.577 (0.019) *	1.780	-0.156 (0.028) *	0.856
At least one year of primary school	0.981 (0.161) *	2.668	0.993 (0.147) *	2.699	-0.425 (0.200) **	0.654
At least one year of secondary school	1.396 (0.162) *	4.039	1.335 (0.149) *	3.801	-0.798 (0.206) *	0.450
At least one year of undergraduate or graduate studies	1.347 (0.168) *	3.847	1.184 (0.154) *	3.268	-0.858 (0.218) *	0.424
Not attending school	-0.256 (0.106) **	0.774	-0.180 (0.098) ***	0.835	0.465 (0.157) *	1.592
Working	0.016 (0.044)	1.016	-0.004 (0.040)	0.996	-0.098 (0.070)	0.906
No walls, dirt, bamboo with mud, cane, palm, trunks, tin, or cardboard	-0.197 (0.084) **	0.821	-0.201 (0.078) *	0.818	0.025 (0.115)	1.025
House or apartment	1.148 (0.315) *	3.153	1.379 (0.304) *	3.970	-0.532 (0.329)	0.587
Electricity	0.289 (0.145) **	1.335	0.280 (0.136) **	1.323	-0.256 (0.175)	0.774
Internet	0.208 (0.050) *	1.232	0.191 (0.045) *	1.210	-0.066 (0.079) ***	0.936
Partner from the same ethnic group	0.213 (0.061) *	1.237	0.172 (0.056) *	1.188	-0.502 (0.085) *	0.605
Afro-descendant or Indigenous individuals	-0.008 (0.157)	0.992	-0.055 (0.143)	0.947	-0.149 (0.170)	0.861
Afro-descendant or Indigenous individuals with a partner from the same ethnic group	-0.304 (0.130) **	0.738	-0.218 (0.120) ***	0.804	0.403 (0.178) **	1.496
Accuracy	0.8046		0.7645		0.9169	
Precision	0.9785		0.9726		0.9991	
Specificity	0.6410		0.6768		0.5000	
Sensitivity	0.8124		0.7703		0.9175	

OR: odds ratio; SE: standard error.

Source: authors’ estimates using the 2015 *Colombian Demography and Health Survey* (DHS) ^19^.

Note: reference categories - women aged from 45 to 49 years, not being a mother, no education, attending school, not working, dwelling different from house or apartment, no electricity, no internet, partners from different ethnic groups, non-ethnic women, and had good wall material. Accuracy = 
$$\frac{P_{11}+P_{00}}{P_{00}+P_{01}+P_{11}+P_{10}}$$

* p-value < 0.01;

** p-value < 0.05;

*** p-value < 0.1.

The odds of using any contraceptive method for women with children were 99% higher than the odds for women without children. This percentage was 78% for the use of modern methods. These results were consistent with the OR of having unmet needs, which was 0.856. In short, women with children have a higher probability of using contraceptive methods and show fewer unmet needs.

Concerning educational level, the comparison category was women with no education. The results indicate that the population group with the highest probability of using contraceptive methods was women with at least one year of secondary education, followed by those with at least one year of undergraduate or graduate studies. This result can be explained by childbearing postponement because women pursued their studies before their family plans ^22^ ^,^ ^27^ ^,^ ^28^ ^,^ ^29^. The result related to missing school was in line with that obtained with educational level: women who were studying were more likely to postpone childbearing and, in this sense, had a higher probability of using contraceptive methods. Moreover, the odds of unmet needs for women outside school were 59% higher than those for women who were studying.

As expected, having a partner from the same ethnic group was associated with a higher probability of using contraceptive methods and with lower probability of unmet needs. However, for afro-descendant and Indigenous women, the association related to these probabilities was inversely related to the association obtained for non-ethnic women. For the estimation of using any contraceptive method (Table 1), the derivatives regarding partner from the same ethnic group report that the coefficient for non-ethnic women is 0.213 and 0.213-0.304 = -0.091 for afro-descendant and Indigenous women. Additionally, based on the derivative regarding afro-descendant and Indigenous women, the OR for for using any contraceptive method and modern methods were 0.732 (calculate as exp[-0.008 -0.304]) and 0.761 (calculated as exp[-0.055 -0.218]), respectively. This means that the odds of these women using contraceptive methods were approximately 24% lower than those of women who did not meet the same conditions regarding ethnic self-identification and partners’ ethnic self-identification. As a result, the OR of having unmet needs between these two groups of women was 1.29 (estimated as exp[-0.149 +0.403]), which means that afro-descendant and Indigenous women women, who usually have partners from the same ethnic group, are less likely than other groups of women to postpone and schedule their pregnancies.

### Family planning methods access and use by ethnic group

**Figure 1:**
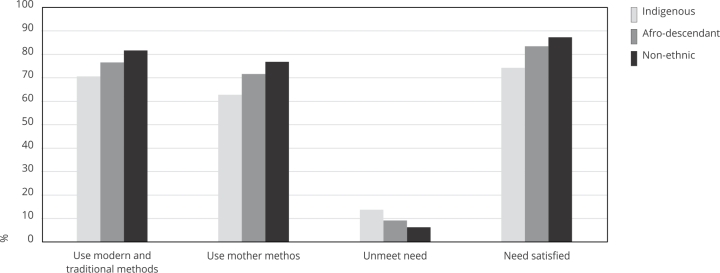
Indicators related to the use of contraceptive methods by ethnic group.

Source: authors’ estimations using the 2015 Colombian Demography and Health Survey (DHS) ^19^ and the 2018 census ^20^. Note: the figures are estimated with the census estimates and weights (see Methods for more information). Non-ethnic refers to all the population who does not self-identify in a specific ethnic group.

Figure 1 compares the results at the national level for the four indicators by ethnic group. The indicators ranking from better to worse is non-ethnic, afro-descendant and Indigenous. The gaps between ethnic groups ranged from three to 14 percentage points, depending on the indicator.

**Figure 2:**
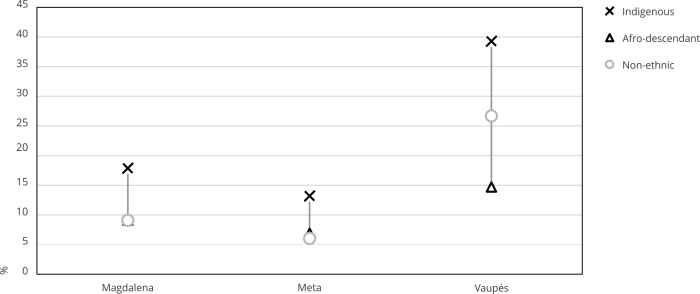
Comparing the proportion of women with unmet need for planning a family between ethnic population.

Source: authors’ estimations using the 2015 Colombian Demography and Health Survey (DHS) ^19^ and 2018 census ^20^. Note: non-ethnic refers to all the population who does not self-identify in a specific ethnic group. The ethnic population consists of individuals who identify themselves as belonging to a particular ethnic group.

Figure 2 contrasts the estimates of the unmet need for family planning between three states with different percentages of afro-descendant and Indigenous women population. There exists a direct relationship between the percentage of afro-descendant and Indigenous women and the gap between the indicators of the different groups. For example, in Vaupés, in which 73% of people identified themselves as belonging to a specific ethnic group, the gap between Indigenous and afro-descendant was approximately 25%. In contrast, in Meta, with a self-identified ethnic population of 2.8%, the gap between these two ethnic groups was 6%. These differences are related to the context in which Indigenous people live since most of them reside in remote rural areas. In contrast, the afro-descendant population is more urban. According to the 2018 Census, in Vaupés, 78% of Indigenous people live in rural areas when compared to 59% of non-ethnics and 25% of afro-descendant individuals. Likewise, for Indigenous, non-ethnic, and afro-descendant individuals, the unmet need for family planning decreased.

**Figure 3:**
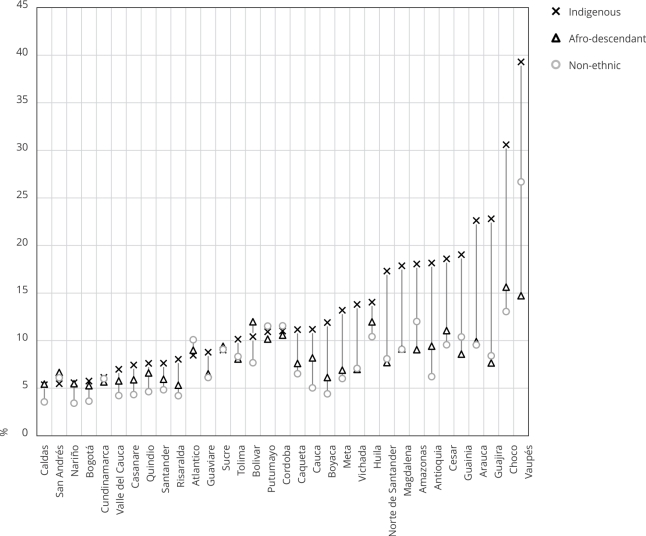
Comparing the unmet need for family planning among ethnic populations and across all states (percentage).

Source: authors’ estimations using the 2015 Colombian Demography and Health Survey (DHS) ^19^ and 2018 census ^20^. Note: non-ethnic refers to all the population who does not self-identify in a specific ethnic group.

Figure 3 compares the unmet family planning needs between ethnic populations across all states. The states are sorted according to the estimated values for their Indigenous population. The 2015 DHS reports that 6.7% of women aged from 15 to 49 years have unmet needs in Colombia. In general, the values for afro-descendant individuals are similar to those for the non-ethnic population toward the right side of the figure and gradually approach the values for Indigenous people toward the left side of the figure. Also, from right to left, the gaps among ethnic groups decrease. 

**Figure 4:**
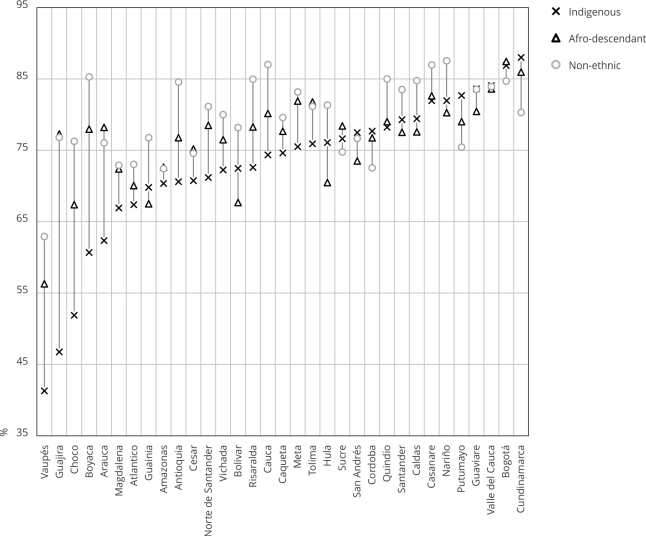
Comparing the contraceptive prevalence rate among ethnic populations and across all states (percentage).

Source: authors’ estimations using the 2015 Colombian Demography and Health Survey (DHS) ^19^ and 2018 census ^20^. Note: non-ethnic refers to all the population who does not self-identify in a specific ethnic group.

Figure 4 shows the results of using modern and traditional methods. As in the previous figure, we find that the values for afro-descendant women are like those for non-ethnic women in the left part of the figure but they align more closely with the values for Indigenous people towards the right part. In fact, in some states, afro-descendant women showed the lowest percentage of contraception method use. In this case, the gaps among ethnic groups decrease from left to right. This might be associated with the urbanicity degree of states: the more urban, the lower the gap.

**Figure 5:**
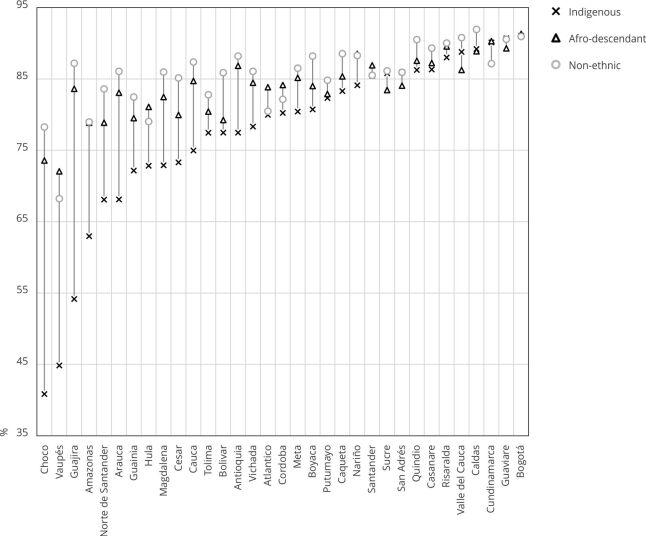
Comparing the need for family planning satisfied with modern methods among ethnic populations and across all states (percentage).

Source: authors’ estimations using the 2015 Colombian Demography and Health Survey (DHS) 19 and 2018 census 20. Note: non-ethnic refers to all the population who does not self-identify in a specific ethnic group.

**Figure 6:**
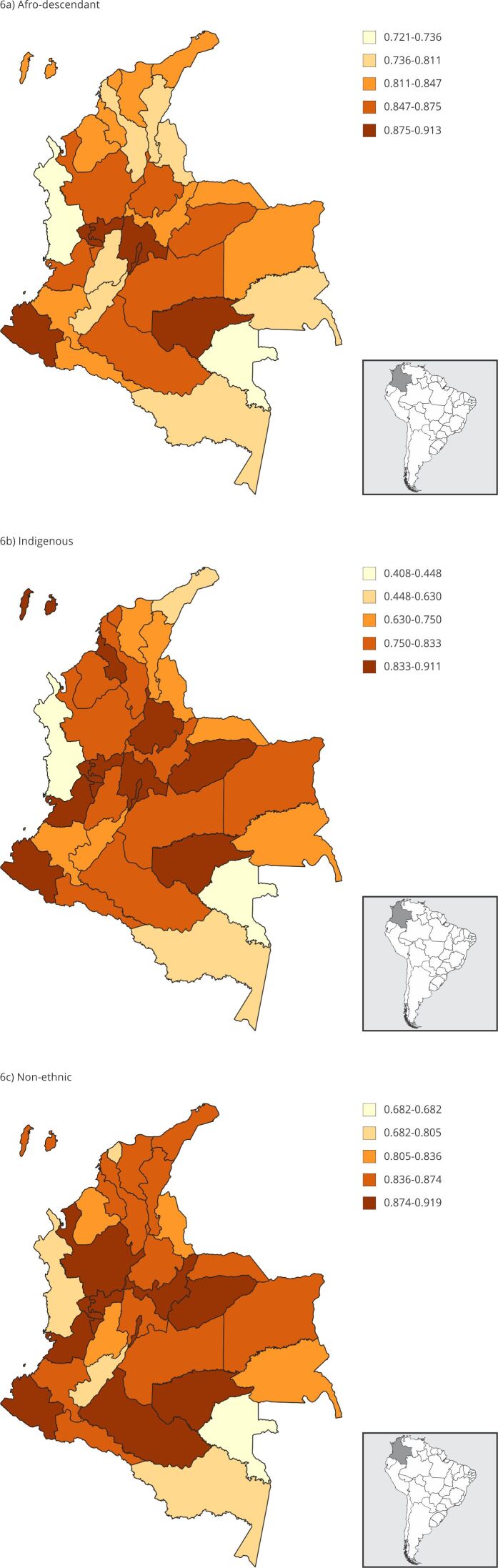
Comparing the need for family planning satisfied with modern methods among ethnic populations and across all states (proportion).

Source: authors’ estimations using the 2015 Colombian Demography and Health Survey (DHS) ^19^ and 2018 census ^20^. Note: non-ethnic refers to all the population that does not self-identify in a specific ethnic group.

Figure 5 shows an interesting result: in the left part of the figure, which mostly lists rural states are listed, the percentage of afro-descendant women who satisfied their family planning needs with modern methods resembles or even exceeds that of non-ethnic women. However, as we move toward the right side of the figure, the percentage for afro-descendant women approaches that of Indigenous women. Figure 6 shows that the percentage of women meeting their needs with modern methods ranged from 40% to 91% for Indigenous women, 72% to 91% for afro-descendant women, and 68% to 92% for non-ethnic women. As ethnic groups mix or interact more with non-ethnic groups, they tend to adopt their customs. As a result, in Bogotá, the three groups show nearly identical percentages of women meeting their family planning needs with modern methods, all averaging 91%.

## Discussion

One major factor to consider when discussing the living conditions of afro-descendant and Indigenous populations, and specially the latter, in Colombia is that they usually live in rural areas with limited access to public services. It is, therefore, essential to highlight that no Internet access is a predominant condition in rural areas with 94.5% of households lacking such access ^21^. However, Internet provision is not the only service that Indigenous communities have problems meeting. Rural women in Colombia have experienced constant inequalities and disadvantages in access to and guarantee of sexual and reproductive rights compared with urban women. These disparities stem from several factors, including difficulty accessing health services in rural areas due to the remote geographical location of many such places, lack of qualified medical personnel to perform different procedures, the presence of armed groups, low schooling levels, lack of economic autonomy, prejudices, little institutional presence, ignorance, and lack of information on contraceptive methods ^30^.

Difficulties in accessing health services for afro-descendant and Indigenous populations are also observed in prenatal care, delivery care, and post-partum monitoring ^31^. Moreover, 69% of households led by an afro-descendant person and 77% of households headed by an Indigenous person experience food insecurity ^32^. The lack of any public services, including health care, added to the severe poor nutrition status reveal a mediating cause between these groups’ living conditions and contraceptive use: infant and child mortality.

Experiencing the death of one’s child negatively affected modern contraception ^33^ ^,^ ^34^. Colombia’s infant mortality rate for males was 18 infants per 1,000 in 2018. For Chocó, where more than 70% of the population are afro-descendants, the rate was 43 per 1,000, and for Vaupés, where more than 70% of residents are Indigenous, it was 30 per 1,000 ^35^. Such high levels of infant and child mortality in ethnic communities may discourage usage of contraceptive methods among afro-descendant and Indigenous women since they have already lost a child or may lose one in the future.

Before controlling for schooling and living conditions, self-identifying as Indigenous or afro-descendants was statistically significant at 90% for contraceptive use and at 95% for unmet needs. After controlling for these variables, however, this self-identification did not decrease the probability of using a contraception method. In other words, it seems that afro-descendant and Indigenous women show a lower prevalence of contraceptive use than non-ethnic women not because of their ethnicity, but rather due to their socio-economic characteristics. However, this finding contrasts with the coefficients of the interaction term “afro-descendant or Indigenous with a partner from the same ethnic group”, which were negative and statistically significant indicating that having a partner from the same ethnic group did decrease these women’s probability of using contraception. In other words, two afro-descendants or Indigenous women with equal schooling and living conditions, but one lives with a non-ethnic partner and the other with an ethnic one, the latter is less likely to use contraceptive methods than the former. 

Cultural traditions may explain these findings. Most afro-descendant and Indigenous peoples are patriarchal and one of their interests is to reproduce. Their prevailing belief is that women are destined for child barring, and the decision to use contraception depends on men ^12^ ^,^ ^14^. Together, these customs and beliefs deprive women of complete autonomy over the number of children they would like to have and may even foster the desire to have as many children as possible. Additionally, polygamy is allowed in several Indigenous groups. Women in polygamist societies tend to believe that being frequently pregnant will increase their husbands’ attention ^33^ ^,^ ^34^ ^,^ ^36^.

Afro-descendant and Indigenous women have limited access to and use of contraceptive methods due to context, partner disapproval, and lack of education ^1^ ^,^ ^2^ ^,^ ^3^ ^,^ ^4^. In short, partner support for contraceptive use is a major factor in increasing usage of contraceptive methods among these women. 

Another key factor is education. Although some ethnic communities are aware of modern contraceptive methods, some erroneous beliefs about their effects on the body persist, creating social barriers to effective access. Some members of such communities believe that modern contraceptive methods can cause illnesses or side effects (e.g., subdermal implants can be lost in the body) and that women (especially teenagers or young adults) who use them do so because they wish to have more than one sexual partner. Additionally, beliefs prevail that vasectomies or condom use cause men to lose their “manhood” or sexual desire ^12^. 

As limitations in our study, we highlight the following associated with data characteristics and selected methodologies. First, we could only integrate questions that were similar or homologous in both the census and the survey. Despite performing validations using univariate analyses, the results obtained are limited to the sample survey.

Second, the DHS 2015 was designed sample-wise to be representative at the rural level for national statistics but not for a subnational level or subgroups. Considering that Colombia’s Indigenous population lives mostly in rural areas of the country, our exercise may have underestimated the measured event. Even with such potential bias, our estimates are important since they help reveal a problem that would most likely be greater if we considered the populations located in more remote areas. Finally, the 3-year difference in data collection is minimal and was compensated by the new cohorts of women entering the evaluated age groups.

We hope that highlighting these limitations will reflect opportunities for new researchers to explore and improve our exercise. For example, authors have proposed machine learning techniques that can complement and improve our exercise. In our case, machine learning techniques could help to leverage more sources of information for the analysis, which could help the accuracy of estimates ^37^ ^,^ ^38^. Likewise, it could improve the segmentation and analysis of population subgroups. We did not explore this methodological option because we opted to obtain coefficients that can be interpreted to be considered for public policy decision-making, as in The Models on the 2015 DHS. We also consider that the greater the simplicity of the methodologies, the greater their comprehensibility and interpretability for policymakers.

## Conclusions

Our article brings three contributions to the literature. First, it illustrates how SAE applicability goes beyond territorial units and can be employed to estimate any indicator for small populations. Second, it measures the access to and use of contraceptive methods among ethnic women which enables analysing any existing gaps. Third, it identifies some of the factors behind these gaps.

Analysing state level gaps reveals how these are perceived in access to modern methods and unmet needs by disaggregating the indicators evaluated by ethnicity or non-ethnicity. Such gaps are double or even triple, primarily associated with the Indigenous and the non-ethnic populations.

Although Indigenous and afro-descendant women have higher fertility rates and lower levels of contraceptive use than non-ethnic ones, our findings indicate that when accounting for variables like schooling, age, and living conditions, being a member of an ethnic group - whether Indigenous or afro-descendant - does not decrease the likelihood of using a contraceptive method. In other words, it appears that afro-descendant and Indigenous women exhibit a lower prevalence of contraceptive use compared with non-ethnic ones not due to their ethnicity, but rather because of their socio-economic characteristics. 

Additionally, being in a relationship increased the likelihood of using contraceptive methods and reduced the likelihood of experiencing unmet needs. However, these two results contrast with the coefficients of the interaction term “afro-descendant or Indigenous with a partner from the same ethnic group”, which indicates that having a partner from the same ethnic group decreases the probability of using a contraception method. In other words, when considering afro-descendant and Indigenous women with partners from the same ethnic group, the associations with the probability of using contraceptive methods differ from those observed in the general case of having a partner. Moreover, afro-descendant and Indigenous women in Colombia tend to form couples with people of the same ethnic group. In the Colombian context, afro-descendant and Indigenous women were also found to form partnerships predominantly within their own ethnic groups - approximately two-thirds, according to the authors’ estimates based on the 2015 DHS. This pattern may be linked to differences in pregnancy planning decisions compared to women from other ethnic backgrounds.

In conclusion, the inequities regarding use of reproductive health services among women from certain ethnic groups in Colombia must be addressed by government strategies that guarantee the right to health. Elaborating these strategies should involve women’s partners and communities.
