# Supplementary material for: Measuring access to contraceptive methods by ethnic groups in Colombia using small areas estimation
Source: Cad Saude Publica. 2025 Jun 27;41(5):e00038024. doi: 10.1590/0102-311XEN038024 (PMC12212300; doi:10.1590/0102-311XEN038024)
Supplement: Supplementary file 1 [file 1678-4464-csp-41-05-EN038024-s.pdf]

Table S1 Use modern and traditional: contraceptive prevalence rate.

State	Ethnic group	Estimated value	Mean square error	Coefficient of variation	Number of people in the 2018 Census
Bogotá	Indigenous	0.86876	0.00319	0.36743	3107
Bogotá	Non-ethnic	0.84701	0.00014	0.01711	915470
Bogotá	Afro-descendant	0.87449	0.00152	0.17375	9364
Bolivar	Indigenous	0.72456	0.00347	0.47939	974
Bolivar	Non-ethnic	0.78185	0.00023	0.02931	229952
Bolivar	Afro-descendant	0.67677	0.00125	0.18446	48553
Boyaca	Indigenous	0.60687	0.00364	0.59917	905
Boyaca	Non-ethnic	0.85292	0.00026	0.03102	141790
Boyaca	Afro-descendant	0.77966	0.00546	0.69989	522
Caldas	Indigenous	0.79423	0.00274	0.34493	6915
Caldas	Non-ethnic	0.84750	0.00037	0.04421	106349
Caldas	Afro-descendant	0.77582	0.00488	0.62894	2002
Caqueta	Indigenous	0.74620	0.00345	0.46201	1261
Caqueta	Non-ethnic	0.79605	0.00029	0.03603	50587
Caqueta	Afro-descendant	0.77672	0.00510	0.65704	695
Cauca	Indigenous	0.74357	0.00128	0.17169	48865
Cauca	Non-ethnic	0.87021	0.00038	0.04336	89397
Cauca	Afro-descendant	0.80162	0.00104	0.13017	33265
Cesar	Indigenous	0.70737	0.00231	0.32684	7412
Cesar	Non-ethnic	0.74587	0.00023	0.03063	134963
Cesar	Afro-descendant	0.75196	0.00195	0.25952	21055
Cordoba	Indigenous	0.77697	0.00104	0.13371	30102
Cordoba	Non-ethnic	0.72545	0.00029	0.04055	182320
Cordoba	Afro-descendant	0.76757	0.00321	0.41845	15394
Cundinamarca	Indigenous	0.88023	0.00309	0.35128	1473
Cundinamarca	Non-ethnic	0.80308	0.00026	0.03263	385572
Cundinamarca	Afro-descendant	0.85958	0.00336	0.39111	2098
Choco	Indigenous	0.51887	0.00136	0.26205	9589
Choco	Non-ethnic	0.76270	0.00103	0.13513	4826
Choco	Afro-descendant	0.67378	0.00031	0.04547	46824
Huila	Indigenous	0.76103	0.00246	0.32282	1831
Huila	Non-ethnic	0.81346	0.00028	0.03426	141066
Huila	Afro-descendant	0.70487	0.00368	0.52244	764
Guajira	Indigenous	0.46738	0.00053	0.11444	55434
Guajira	Non-ethnic	0.76807	0.00047	0.06110	52780
Guajira	Afro-descendant	0.77297	0.00237	0.30639	8238
Magdalena	Indigenous	0.66913	0.00345	0.51579	3031
Magdalena	Non-ethnic	0.72905	0.00022	0.03077	168151
Magdalena	Afro-descendant	0.72393	0.00153	0.21119	15982
Antioquia	Indigenous	0.70602	0.00243	0.34460	5854
Antioquia	Non-ethnic	0.84581	0.00012	0.01452	711074
Antioquia	Afro-descendant	0.76771	0.00129	0.16796	43995
Meta	Indigenous	0.75512	0.00356	0.47171	3335
Meta	Non-ethnic	0.83187	0.00027	0.03231	129060
Meta	Afro-descendant	0.81911	0.00422	0.51518	1222
Nariño	Indigenous	0.81964	0.00082	0.10021	27665
Nariño	Non-ethnic	0.87558	0.00056	0.06364	103351
Nariño	Afro-descendant	0.80281	0.00098	0.12167	32311
Norte de Santander	Indigenous	0.71187	0.00435	0.61147	647
Norte de Santander	Non-ethnic	0.81150	0.00029	0.03522	187301
Norte de Santander	Afro-descendant	0.78504	0.00444	0.56618	719
Quindio	Indigenous	0.78240	0.00432	0.55156	440
Quindio	Non-ethnic	0.85007	0.00032	0.03734	61647
Quindio	Afro-descendant	0.79018	0.00354	0.44818	783
Risaralda	Indigenous	0.72601	0.00345	0.47530	4514
Risaralda	Non-ethnic	0.84967	0.00037	0.04324	102358
Risaralda	Afro-descendant	0.78265	0.00344	0.43913	2170
Santander	Indigenous	0.79307	0.00481	0.60713	198
Santander	Non-ethnic	0.83511	0.00020	0.02429	274701
Santander	Afro-descendant	0.77528	0.00444	0.57295	3207
Sucre	Indigenous	0.76621	0.00109	0.14183	16294
Sucre	Non-ethnic	0.74771	0.00024	0.03154	94400
Sucre	Afro-descendant	0.78407	0.00292	0.37182	14803
Tolima	Indigenous	0.75913	0.00130	0.17060	6379
Tolima	Non-ethnic	0.81138	0.00038	0.04652	153205
Tolima	Afro-descendant	0.81785	0.00381	0.46626	708

Valle del Cauca	Indigenous	0.84091	0.00299	0.35565	5111
Valle del Cauca	Non-ethnic	0.83942	0.00020	0.02360	405672
Valle del Cauca	Afro-descendant	0.83599	0.00051	0.06055	86238
Atlantico	Indigenous	0.67382	0.00316	0.46843	6126
Atlantico	Non-ethnic	0.73037	0.00020	0.02685	316990
Atlantico	Afro-descendant	0.70046	0.00177	0.25301	20847
Arauca	Indigenous	0.62354	0.00360	0.57761	946
Arauca	Non-ethnic	0.76033	0.00043	0.05602	34147
Arauca	Afro-descendant	0.78228	0.00358	0.45760	1526
Casanare	Indigenous	0.81956	0.00450	0.54964	1032
Casanare	Non-ethnic	0.86963	0.00033	0.03770	56992
Casanare	Afro-descendant	0.82627	0.00391	0.47288	893
Putumayo	Indigenous	0.82691	0.00171	0.20728	7227
Putumayo	Non-ethnic	0.75432	0.00047	0.06190	30745
Putumayo	Afro-descendant	0.79015	0.00318	0.40215	1274
San Andrés	Indigenous	0.77493	0.04707	6.07382	3
San Andrés	Non-ethnic	0.76664	0.00030	0.03879	3098
San Andrés	Afro-descendant	0.73506	0.00133	0.18038	882
Amazonas	Indigenous	0.70356	0.00048	0.06796	5277
Amazonas	Non-ethnic	0.72435	0.00075	0.10415	3407
Amazonas	Afro-descendant	0.72638	0.00614	0.84552	46
Guainia	Indigenous	0.69814	0.00061	0.08707	4443
Guainia	Non-ethnic	0.76771	0.00108	0.14045	1269
Guainia	Afro-descendant	0.67499	0.00772	1.14417	54
Guaviare	Indigenous	0.83628	0.00257	0.30743	965
Guaviare	Non-ethnic	0.83544	0.00036	0.04251	8925
Guaviare	Afro-descendant	0.80446	0.00381	0.47406	366
Vaupes	Indigenous	0.41297	0.00037	0.08911	3376
Vaupes	Non-ethnic	0.62925	0.00111	0.17565	493
Vaupes	Afro-descendant	0.56297	0.00874	1.55261	29
Vichada	Indigenous	0.72253	0.00108	0.14971	6347
Vichada	Non-ethnic	0.80001	0.00037	0.04657	4428
Vichada	Afro-descendant	0.76487	0.00575	0.75218	83

Source: Authors' estimations with the 2018-Census and 2015-DHS. Notes: the Coefficient of variation is the Mean square error over the Estimated value multiplied by 100.

Table S2 Use modern: modern contraceptive prevalence rate.

State	Ethnic group	Estimated value	Mean square error	Coefficient of variation	Number of people in the 2018 Census
Bogotá	Indigenous	0.78015	0.83857	0.00347	3107
Bogotá	Non-ethnic	0.75745	0.80333	0.00017	915470
Bogotá	Afro-descendant	0.80268	0.84409	0.00167	9364
Bolivar	Indigenous	0.60600	0.64738	0.00374	974
Bolivar	Non-ethnic	0.71015	0.73780	0.00024	229952
Bolivar	Afro-descendant	0.59209	0.62934	0.00131	48553
Boyaca	Indigenous	0.30632	0.47925	0.00276	905
Boyaca	Non-ethnic	0.76230	0.79136	0.00031	141790
Boyaca	Afro-descendant	0.65918	0.70521	0.00547	522
Caldas	Indigenous	0.67424	0.74294	0.00283	6915
Caldas	Non-ethnic	0.78593	0.81289	0.00044	106349
Caldas	Afro-descendant	0.67027	0.72886	0.00520	2002
Caqueta	Indigenous	0.58261	0.69115	0.00357	1261
Caqueta	Non-ethnic	0.71994	0.76328	0.00028	50587
Caqueta	Afro-descendant	0.68218	0.72901	0.00493	695
Cauca	Indigenous	0.46406	0.63719	0.00125	48865
Cauca	Non-ethnic	0.76344	0.80899	0.00046	89397
Cauca	Afro-descendant	0.66804	0.74956	0.00120	33265
Cesar	Indigenous	0.57103	0.66026	0.00244	7412
Cesar	Non-ethnic	0.68138	0.71611	0.00030	134963
Cesar	Afro-descendant	0.65401	0.69601	0.00219	21055
Cordoba	Indigenous	0.66611	0.71543	0.00106	30102
Cordoba	Non-ethnic	0.65671	0.69053	0.00037	182320
Cordoba	Afro-descendant	0.70430	0.73569	0.00361	15394
Cundinamarca	Indigenous	0.81248	0.84481	0.00370	1473
Cundinamarca	Non-ethnic	0.70468	0.75203	0.00030	385572
Cundinamarca	Afro-descendant	0.78981	0.82654	0.00331	2098
Choco	Indigenous	0.27624	0.35830	0.00155	9589
Choco	Non-ethnic	0.64529	0.69897	0.00112	4826
Choco	Afro-descendant	0.55612	0.60975	0.00032	46824
Huila	Indigenous	0.60974	0.66879	0.00273	1831
Huila	Non-ethnic	0.69791	0.72500	0.00030	141066
Huila	Afro-descendant	0.62432	0.66036	0.00413	764
Guajira	Indigenous	0.28490	0.38084	0.00050	55434
Guajira	Non-ethnic	0.70105	0.74360	0.00053	52780
Guajira	Afro-descendant	0.67228	0.71647	0.00258	8238
Magdalena	Indigenous	0.48322	0.59957	0.00327	3031
Magdalena	Non-ethnic	0.66325	0.70523	0.00028	168151
Magdalena	Afro-descendant	0.62480	0.67645	0.00165	15982
Antioquia	Indigenous	0.50797	0.65114	0.00225	5854
Antioquia	Non-ethnic	0.77598	0.80166	0.00015	711074
Antioquia	Afro-descendant	0.69085	0.74299	0.00157	43995
Meta	Indigenous	0.50251	0.67590	0.00326	3335
Meta	Non-ethnic	0.73089	0.77239	0.00034	129060
Meta	Afro-descendant	0.70292	0.75674	0.00401	1222
Nariño	Indigenous	0.62547	0.72877	0.00082	27665
Nariño	Non-ethnic	0.76668	0.80464	0.00065	103351
Nariño	Afro-descendant	0.66767	0.76288	0.00114	32311
Norte de Santander	Indigenous	0.47541	0.60005	0.00396	647
Norte de Santander	Non-ethnic	0.71258	0.74598	0.00030	187301
Norte de Santander	Afro-descendant	0.64208	0.68437	0.00464	719
Quindio	Indigenous	0.58638	0.71623	0.00428	440
Quindio	Non-ethnic	0.77687	0.81137	0.00036	61647
Quindio	Afro-descendant	0.67776	0.74758	0.00388	783
Risaralda	Indigenous	0.54029	0.68470	0.00339	4514
Risaralda	Non-ethnic	0.77824	0.80326	0.00042	102358
Risaralda	Afro-descendant	0.71305	0.75221	0.00368	2170
Santander	Indigenous	0.64305	0.73092	0.00478	198
Santander	Non-ethnic	0.72213	0.75553	0.00027	274701
Santander	Afro-descendant	0.68157	0.72535	0.00494	3207
Sucre	Indigenous	0.68017	0.73349	0.00125	16294
Sucre	Non-ethnic	0.68123	0.72285	0.00029	94400
Sucre	Afro-descendant	0.68548	0.73414	0.00325	14803
Tolima	Indigenous	0.63067	0.66970	0.00138	6379
Tolima	Non-ethnic	0.71665	0.74044	0.00043	153205
Tolima	Afro-descendant	0.69864	0.72661	0.00400	708

Valle del Cauca	Indigenous	0.72351	0.80273	0.00315	5111
Valle del Cauca	Non-ethnic	0.76971	0.80180	0.00023	405672
Valle del Cauca	Afro-descendant	0.72430	0.77281	0.00058	86238
Atlantico	Indigenous	0.56779	0.60845	0.00351	6126
Atlantico	Non-ethnic	0.62999	0.66938	0.00021	316990
Atlantico	Afro-descendant	0.61710	0.66131	0.00198	20847
Arauca	Indigenous	0.48725	0.57637	0.00338	946
Arauca	Non-ethnic	0.71087	0.73736	0.00047	34147
Arauca	Afro-descendant	0.70624	0.73472	0.00400	1526
Casanare	Indigenous	0.60064	0.74956	0.00431	1032
Casanare	Non-ethnic	0.77363	0.81570	0.00035	56992
Casanare	Afro-descendant	0.70811	0.77045	0.00431	893
Putumayo	Indigenous	0.69355	0.78149	0.00186	7227
Putumayo	Non-ethnic	0.65348	0.73673	0.00057	30745
Putumayo	Afro-descendant	0.66300	0.74980	0.00350	1274
San Andrés	Indigenous	0.70167	0.71514	0.05804	3
San Andrés	Non-ethnic	0.69230	0.71119	0.00036	3098
San Andrés	Afro-descendant	0.65089	0.67350	0.00141	882
Amazonas	Indigenous	0.59177	0.56122	0.00054	5277
Amazonas	Non-ethnic	0.66340	0.64763	0.00081	3407
Amazonas	Afro-descendant	0.64930	0.63352	0.00669	46
Guainia	Indigenous	0.56063	0.64476	0.00070	4443
Guainia	Non-ethnic	0.67562	0.71683	0.00124	1269
Guainia	Afro-descendant	0.57788	0.61210	0.00847	54
Guaviare	Indigenous	0.62538	0.82353	0.00239	965
Guaviare	Non-ethnic	0.72396	0.81235	0.00041	8925
Guaviare	Afro-descendant	0.64326	0.77744	0.00403	366
Vaupes	Indigenous	0.52997	0.36246	0.00041	3376
Vaupes	Non-ethnic	0.66543	0.55376	0.00128	493
Vaupes	Afro-descendant	0.60208	0.49158	0.00908	29
Vichada	Indigenous	0.48881	0.66758	0.00110	6347
Vichada	Non-ethnic	0.70247	0.75720	0.00040	4428
Vichada	Afro-descendant	0.65303	0.71459	0.00655	83

Source: Authors' estimations with the 2018-Census and 2015-DHS. Notes: the Coefficient of variation is the Mean square error over the Estimated value multiplied by 100.

Table S3 Unmet need: proportion of women with unmet need for family planning.

State	Ethnic group	Estimated value	Mean square error	Coefficient of variation	Number of people in the 2018 Census
Bogotá	Indigenous	0.05746	0.00051	0.89523	3107
Bogotá	Non-ethnic	0.03631	0.00005	0.12923	915470
Bogotá	Afro-descendant	0.05256	0.00037	0.69919	9364
Bolivar	Indigenous	0.10412	0.00066	0.63136	974
Bolivar	Non-ethnic	0.07682	0.00011	0.14826	229952
Bolivar	Afro-descendant	0.11979	0.00035	0.29120	48553
Boyaca	Indigenous	0.11903	0.00384	3.22915	905
Boyaca	Non-ethnic	0.04403	0.00008	0.19252	141790
Boyaca	Afro-descendant	0.06117	0.00065	1.07069	522
Caldas	Indigenous	0.05365	0.00071	1.32267	6915
Caldas	Non-ethnic	0.03557	0.00013	0.36647	106349
Caldas	Afro-descendant	0.05426	0.00070	1.28922	2002
Caqueta	Indigenous	0.11158	0.00159	1.42198	1261
Caqueta	Non-ethnic	0.06523	0.00016	0.25111	50587
Caqueta	Afro-descendant	0.07596	0.00078	1.03144	695
Cauca	Indigenous	0.11180	0.00052	0.46915	48865
Cauca	Non-ethnic	0.05028	0.00019	0.36955	89397
Cauca	Afro-descendant	0.08184	0.00046	0.56693	33265
Cesar	Indigenous	0.18591	0.00149	0.79881	7412
Cesar	Non-ethnic	0.09556	0.00010	0.10845	134963
Cesar	Afro-descendant	0.11054	0.00046	0.41281	21055
Cordoba	Indigenous	0.11017	0.00053	0.47920	30102
Cordoba	Non-ethnic	0.11558	0.00013	0.11482	182320
Cordoba	Afro-descendant	0.10571	0.00087	0.82607	15394
Cundinamarca	Indigenous	0.06133	0.00048	0.78292	1473
Cundinamarca	Non-ethnic	0.06006	0.00008	0.12570	385572
Cundinamarca	Afro-descendant	0.05659	0.00045	0.79597	2098
Choco	Indigenous	0.30597	0.00134	0.43848	9589
Choco	Non-ethnic	0.13059	0.00056	0.42730	4826
Choco	Afro-descendant	0.15616	0.00022	0.14385	46824
Huila	Indigenous	0.14045	0.00076	0.53875	1831
Huila	Non-ethnic	0.10406	0.00010	0.09575	141066
Huila	Afro-descendant	0.11972	0.00046	0.38489	764
Guajira	Indigenous	0.22804	0.00080	0.34888	55434
Guajira	Non-ethnic	0.08400	0.00023	0.27416	52780
Guajira	Afro-descendant	0.07649	0.00046	0.60509	8238
Magdalena	Indigenous	0.17870	0.00265	1.48508	3031
Magdalena	Non-ethnic	0.09078	0.00013	0.14099	168151
Magdalena	Afro-descendant	0.09130	0.00050	0.54478	15982
Antioquia	Indigenous	0.18163	0.00164	0.90085	5854
Antioquia	Non-ethnic	0.06210	0.00006	0.10075	711074
Antioquia	Afro-descendant	0.09420	0.00038	0.40222	43995
Meta	Indigenous	0.13184	0.00219	1.66156	3335
Meta	Non-ethnic	0.06013	0.00011	0.18115	129060
Meta	Afro-descendant	0.06893	0.00049	0.71786	1222
Nariño	Indigenous	0.05570	0.00056	1.00575	27665
Nariño	Non-ethnic	0.03429	0.00021	0.60337	103351
Nariño	Afro-descendant	0.05473	0.00056	1.02233	32311
Norte de Santander	Indigenous	0.17304	0.00235	1.35649	647
Norte de Santander	Non-ethnic	0.08086	0.00012	0.15004	187301
Norte de Santander	Afro-descendant	0.07687	0.00057	0.74612	719
Quindio	Indigenous	0.07606	0.00133	1.74398	440
Quindio	Non-ethnic	0.04628	0.00013	0.27550	61647
Quindio	Afro-descendant	0.06605	0.00057	0.86356	783
Risaralda	Indigenous	0.08030	0.00181	2.24895	4514
Risaralda	Non-ethnic	0.04211	0.00011	0.25000	102358
Risaralda	Afro-descendant	0.05310	0.00038	0.70959	2170
Santander	Indigenous	0.07616	0.00120	1.57126	198
Santander	Non-ethnic	0.04836	0.00008	0.17127	274701
Santander	Afro-descendant	0.05939	0.00048	0.81457	3207
Sucre	Indigenous	0.09001	0.00044	0.49092	16294
Sucre	Non-ethnic	0.09076	0.00011	0.11964	94400
Sucre	Afro-descendant	0.09396	0.00054	0.57297	14803
Tolima	Indigenous	0.10153	0.00045	0.43976	6379
Tolima	Non-ethnic	0.08330	0.00012	0.14588	153205
Tolima	Afro-descendant	0.08062	0.00052	0.64289	708

Valle del Cauca	Indigenous	0.06980	0.00092	1.31169	5111
Valle del Cauca	Non-ethnic	0.04216	0.00009	0.20268	405672
Valle del Cauca	Afro-descendant	0.05761	0.00016	0.26942	86238
Atlantico	Indigenous	0.08464	0.00042	0.50052	6126
Atlantico	Non-ethnic	0.10088	0.00007	0.06934	316990
Atlantico	Afro-descendant	0.08986	0.00045	0.49949	20847
Arauca	Indigenous	0.22624	0.00211	0.93322	946
Arauca	Non-ethnic	0.09551	0.00016	0.16591	34147
Arauca	Afro-descendant	0.09899	0.00068	0.68830	1526
Casanare	Indigenous	0.07437	0.00139	1.87304	1032
Casanare	Non-ethnic	0.04334	0.00014	0.31265	56992
Casanare	Afro-descendant	0.05877	0.00063	1.07789	893
Putumayo	Indigenous	0.10934	0.00066	0.60769	7227
Putumayo	Non-ethnic	0.11527	0.00019	0.16736	30745
Putumayo	Afro-descendant	0.10155	0.00065	0.63574	1274
San Andrés	Indigenous	0.05469	0.01344	24.58458	3
San Andrés	Non-ethnic	0.06099	0.00013	0.20702	3098
San Andrés	Afro-descendant	0.06656	0.00032	0.48011	882
Amazonas	Indigenous	0.18048	0.00035	0.19489	5277
Amazonas	Non-ethnic	0.12008	0.00038	0.31298	3407
Amazonas	Afro-descendant	0.09039	0.00155	1.71536	46
Guainia	Indigenous	0.19039	0.00054	0.28444	4443
Guainia	Non-ethnic	0.10381	0.00032	0.31267	1269
Guainia	Afro-descendant	0.08578	0.00161	1.87661	54
Guaviare	Indigenous	0.08783	0.00125	1.42003	965
Guaviare	Non-ethnic	0.06115	0.00020	0.33395	8925
Guaviare	Afro-descendant	0.06503	0.00088	1.35590	366
Vaupes	Indigenous	0.39295	0.00040	0.10121	3376
Vaupes	Non-ethnic	0.26692	0.00044	0.16453	493
Vaupes	Afro-descendant	0.14714	0.00324	2.20064	29
Vichada	Indigenous	0.13797	0.00124	0.89704	6347
Vichada	Non-ethnic	0.07084	0.00018	0.25982	4428
Vichada	Afro-descendant	0.06978	0.00146	2.08516	83

Source: Authors' estimations with the 2018-Census and 2015-DHS. Notes: the Coefficient of variation is the Mean square error over the Estimated value multiplied by 100.
